# Intracellular Localization of *Blattella germanica* Densovirus (BgDV1) Capsid Proteins

**DOI:** 10.3390/v10070370

**Published:** 2018-07-14

**Authors:** Evgeny N. Kozlov, Elena U. Martynova, Vladimir I. Popenko, Coby Schal, Dmitry V. Mukha

**Affiliations:** 1 Vavilov Institute of General Genetics Russian Academy of Sciences, 119991 Moscow, Russia; ugin.sfu@gmail.com (E.N.K.); elenamartynovaster@gmail.com (E.U.M.); 2 Engelhardt Institute of Molecular Biology Russian Academy of Sciences, 119991 Moscow, Russia; popenko@eimb.ru; 3 Department of Entomology and Plant Pathology, North Carolina State University, Raleigh, NC 27695-7613, USA; coby@ncsu.edu

**Keywords:** Parvoviridae, Densovirinae, *Blattella germanica* densovirus, BgDV1, capsid proteins, nuclear localization, nuclear export, NLS, NES

## Abstract

Densovirus genome replication and capsid assembly take place in the nucleus of the infected cells. However, the mechanisms underlying such processes as the delivery of virus proteins to the nucleus and the export of progeny virus from the nucleus remain elusive. It is evident that nuclear transport signals should be involved in these processes. We performed an in silico search for the putative nuclear localization signal (NLS) and nuclear export signal (NES) motifs in the capsid proteins of the *Blattella germanica* Densovirus 1 (BgDV1) densovirus. A high probability NLS motif was found in the common C-terminal of capsid proteins together with a NES motif in the unique N-terminal of VP2. We also performed a global search for the nuclear traffic signals in the densoviruses belonging to five Densovirinae genera, which revealed high diversity in the patterns of NLSs and NESs. Using a heterologous system, the HeLa mammalian cell line expressing GFP-fused BgDV1 capsid proteins, we demonstrated that both signals are functionally active. We suggest that the NLS shared by all three BgDV1 capsid proteins drives the trafficking of the newly-synthesized proteins into the nucleus, while the NES may play a role in the export of the newly-assembled BgDV1 particles into the cytoplasm through nuclear pore complexes.

## 1. Introduction

Densoviruses comprise the subfamily Densovirinae of invertebrate-infecting viruses within the family Parvoviridae. Another subfamily in the Parvoviridae, the Parvovirinae, contains viruses infecting vertebrates, including birds, mammals, and humans. Densoviruses represent a highly diversified group in terms of their genome size, organization and expression strategies, which is at present subdivided into five genera Ambidensovirus, *Brevidensovirus*, *Hepandensovirus*, *Iteradensovirus*, and *Penstyldensovirus* [1]. Densoviruses are generally characterized by a small non-enveloped icosahedral capsid 18–26 nm in diameter and a linear single-stranded DNA genome 4–6.5 kb in size, encoding two sets of proteins, the regulatory (NS) and capsid (VP) proteins [2,3].

Densovirus capsids are rather simply organized, being made up of 60 capsid protein subunits [4]. Densoviruses possess from two to five capsid proteins depending on the genus to which they belong: four capsid proteins in lepidopteran members of *Ambidensovirus* and *Acheta domestica* densovirus (AdDV) [5,6,7,8,9]; three or four capsid proteins in *Culex pipiens* densovirus (CpDV) [10]; five proteins in *Periplaneta fuliginosa* densovirus (PfDV) [11,12]; two or three proteins in *Brevidensovirus* [13]; four proteins in *Penstyldensvirus* [14]; four or five proteins in *Iteradensovirus* [15,16,17], and one or two proteins in *Hepandensovirus* [18]. Remarkable features of densovirus capsid proteins are that they share common C-terminal regions corresponding to the entire smallest capsid protein, and that the larger proteins possess unique N-terminal residues which play essential roles during the virus life cycle. For example, the unique N-terminal region of the largest protein in all densoviruses, VP1, contains the PLA_2_ motif which is indispensable for the virus to escape from the late endosome during its trafficking to the nucleus [5,15,17,19], with the only exception being the representatives of the genera *Brevidensovirus*, *Penstyldensovirus*, and *Hepandensovirus* [3]. The smallest capsid protein is, in most cases, the major structural unit of the densovirus capsid, with the larger proteins being present in just minor quantities [2]. Densovirus capsids perform multiple functional roles, including host cell surface receptor recognition, pathogenicity determination, self-assembly into capsids, virus genetic material delivery, escape from the late endosome during infection, and nuclear import and export [4].

While it has been amply demonstrated that densovirus genome replication and capsid assembly take place in the nucleus of the infected cells [2,3], the mechanisms underlying such processes as the delivery of virus proteins to the nucleus and the export of the progeny virus out of the nucleus remain elusive. It is evident that nuclear transport signals, such as nuclear localization signals (NLSs) and nuclear export signals (NESs) should be involved in these processes.

NLSs usually consist of one or more short sequences of positively charged lysines or arginines exposed on the protein surface. The NLSs are subdivided into classical and nonclassical NLSs. Classical NLSs can be subdivided further into two types, the monopartite and bipartite NLSs. The former are highly basic and consist of a short stretch of basic amino acids and are exemplified first of all by the NLS of the SV40 large T-antigen PKKKRKV [20] and c-myc NLS PAAKRVKLD [21], as well as some other viral and nonviral NLSs. Kosugi et al. [22] described six classes of NLSs, of which the classical monopartite NLSs bind to the major binding pocket of importin alpha, i.e., SV40 large T-antigen-like NLSs as class 1 and c-myc-like NLS as class 2 NLSs. The bipartite motif consists of two stretches of basic amino acids which are separated by a 10–12 amino acid spacer [22,23]. This motif is exemplified by the NLS of nucleoplasmin KRPAATKKAGQAKKKK and requires the regions outside the core basic residues for its activity [24,25]. There also exist a significant number of NLS motifs that do not fit within the consensus sequences of the classical NLSs, such as arginine-rich NLSs, proline-tyrosine (P-Y) NLSs, human hnRNP A1, and other atypical NLSs [26,27], and they are therefore referred to as nonclassical, or noncanonical, NLSs. It should be noted that in bioinformatic predictions, two motifs are usually used to detect potential monopartite NLSs within a protein sequence: the Pat4 motif, which consists of four continuous basic amino acids, and the Pat7 motif, which consists of a proline followed by three out of four basic residues after a stretch of one to three amino acids [P-X(1-3)-(3-4K/R)] [28], the former referring to class I and the latter to class II NLSs. These motifs also correspond with the consensus sequence K(R/K)X(R/K) (X − K, R, P, V, A) for an efficient NLS suggested by Chelsky et al. [29]. The third motif used in bioinformatic predictions is the classical bipartite motif [28].

A nuclear export signal (NES) is a short amino acid sequence of four hydrophobic residues that targets the protein for export from the cell nucleus to the cytoplasm through the nuclear pore complex. The NES is currently defined as a peptide that is 8–15 residues long and conforms loosely to the widely used traditional consensus of Φ1-X2,3-Φ2-X2,3-Φ3-X-Φ4, where Φn represents Leu, Val, Ile, Phe, or Met, and X can be any amino acid [30].

Densoviruses as well as vertebrate parvoviruses exploit the host cell polymerase to reconstitute the double stranded genome prior to transcription and to produce progeny with single-stranded DNA genomes. For this reason, they need cells in the S-phase of the cell cycle, most often rapidly dividing cells, with viral DNA replication, viral particle assembly, and viral genome encapsidation taking place in the nucleus [3]. However, little is known about how the densovirus viral particles reach and enter the nucleus and how the progeny virions exit the nucleus to accumulate in the cytoplasm. It has been demonstrated by Vendeville et al. [31] that similar to vertebrate parvoviruses, JcDV rapidly enters the cell by dynamin-dependent clathrin-mediated endocytosis. The rapid entry is followed by slower transfer through the system of endosomes, first into the early endosomes, then into the late endosomes which are found in the perinuclear compartment [31]. It was observed that the PLA2-containing part of the capsid protein is externalized through the channel at the capsid’s 5-fold axis which enables the virion to breach the endosomal membrane [4]. Since many stages in the densovirus life cycle are tightly associated with the nuclear compartment, it was suggested that strong NLSs should be associated with densovirus proteins, including capsid proteins, which may contribute to nuclear entry of the virus particles, and newly synthesized viral proteins through the nuclear pore complex [27].

It has been in fact demonstrated that in vertebrate parvoviruses, capsid proteins contain basic amino acid clusters that function as NLSs, both in the unique region of the largest capsid protein, and in the common C-terminal region shared by all capsid proteins [28,32,33,34,35,36,37]. The identified NLSs are both canonical and non-canonical, including structural nuclear localization motifs [28]. These signals play an essential role in the parvovirus life cycle, directing the nuclear import of virus particles following entry and escape from the endosome [4,28] and of viral capsid proteins/protein complexes for subsequent capsid assembly and viral progeny production [38]. However, the precise roles for each identified NLS, as well as their functionality, have yet to be established. It was also suggested that NLS may be required for normal viral capsid assembly in the nucleus of infected cells. Furthermore, it has been demonstrated that mutations in the BR4 nuclear localization motif in AAV2 may affect early stages of virus assembly or virus trafficking [33]. Current knowledge on the NLSs identified in the structural proteins of parvoviruses is extensively discussed in a recent review by Liu et al. [38].

In densoviruses, the presence of NLS motifs has been so far predicted only for a few representatives of the group, including the NLSs in capsid and regulatory proteins of penaeid densoviruses [27], NLSs are present in the capsid proteins of the densoviruses from *Aedes aegypti* and *Aedes albopictus* [39,40], and in the main regulatory NS1 protein of PfDV [41]. In the latter case, the functional activity of the corresponding signal has been experimentally demonstrated.

Little is known on how the newly-assembled densovirus and parvovirus particles egress from the nucleus. It should be noted that densoviruses, and parvoviruses in general, are among the smallest viruses described so far. The small virus particle size, which is comparable with the size of the nuclear pore, suggests that the progeny virus particles may be transferred from the nucleus through the nuclear pore, avoiding nuclear membrane disruption, and some evidence supports this idea [42,43]. It was also suggested that NESs may be involved in this process; however, currently available evidence on the precise roles of NESs in the parvovirus life cycle is rather scarce. A study performed using the Minute Virus of Mice (MVM) demonstrated that MVM virions can actively exit the nucleus [44]. The unique N-terminus of the major capsid protein VP2 (2Nt) was essentially required as a signal for trafficking capsids out of the nucleus. Another critical factor was the phosphorylation level of the serine residues within 2Nt. Highly phosphorylated 2Nt drove nuclear export through the noncanonical Exportin-1-independent pathway, while the nonphosphorylated 2Nt may switch it to the exportin-1-dependent pathway [44]. Interestingly, a noncanonical NES was found in the MVM regulatory protein NS2, and it has been demonstrated that this motif is recognized by Exportin-1, the major receptor participating in the protein export out of the nucleus [45], and exported from the nucleus according to the Exportin-1-dependent mechanism. Moreover, it has been further shown that the interaction between NS2 and the nuclear export receptor is critical for the release of progeny viruses [46,47,48,49]. In regards to NES motifs, no such signals have so far been predicted in any densovirus proteins.

The densovirus of the German cockroach *Blattella germanica* (BgDV1) was first described in 2000 in a laboratory-maintained cockroach line, P6 [50]. The expression strategy of the 5335 nt antisense BgDV1 genome has been thoroughly investigated [51]. In particular, it was demonstrated that the three capsid proteins VP1, VP2, and VP3 of BgDV1 with molecular weights of 100 kDa, 80/85 kDa, and 56 kDa, respectively, are translated from two messenger RNAs (mRNAs). The unspliced mRNA encodes VP2 and the spliced transcript contains a single open reading frame resulting from the joining of the two open reading frames for capsid proteins, which is translated into VP1 and VP3, likely using the “leaky-scanning” mechanism. As is the case with all densoviruses, the VP3 protein is contained in both VP1 and VP2 proteins at their common C-terminal. The unique N-terminal of VP1 contains the conserved PLA_2_ motif.

In our pilot experiments, the intracellular localization of the fusion protein composed of green fluorescent protein (GFP) and one of the capsid proteins (namely VP1) of BgDV1 was investigated using the HeLa human cell culture. The intracellular localization of GFP was analyzed in a series of control experiments. Histochemical analysis showed that the fusion protein was localized exclusively inside the nucleus of cells because of the transitory expression of the corresponding vector constructs, whereas the GFP was located both in the nucleus and the cytoplasm [52]. Moreover, using Western blot analysis of nuclear and cytoplasmic extracts of cells infected by BgDV1, the intracellular localization of the regulatory proteins of the densovirus was investigated. It was demonstrated that two proteins, namely NS1 and NS3, were predominantly localized in the nucleus, whereas the regulatory protein NS2 was equally distributed in the nuclei and the cytoplasm [53]. These observations extended the experimental possibilities for studying the genetic control of intracellular traffic of densovirus proteins.

In this paper, we report the search for potential intracellular traffic signals in the BgDV1 capsid proteins VP1, VP2, and VP3. Our in silico analysis revealed the presence of NLS motifs in all three BgDV1 capsid proteins, as well as NES motif in the unique N-terminal region of the VP2 protein. Using the same approach as with BgDV1, we performed a global search for the intracellular traffic signals in representatives of all five Densovirinae genera, which demonstrated that capsid proteins of all analyzed densoviruses contain NLS motifs (although the probability score of the detected NLSs varied significantly among different types of densoviruses), whereas NES motifs were predicted in most densoviruses with the exception for the lepidopteran genera *Ambidensovirus*, *Iteravirus*, and *Penstyldensovirus*. We tested the functional activity of the predicted NLS and NES motifs in the BgDV1 capsid proteins. By introducing amino acid substitutions in the in silico-predicted intracellular traffic signals, we experimentally demonstrated that both the high-probability NLS and NES are functionally active and mediate respectively the nuclear import and the nuclear export of BgDV1 capsid proteins. We also demonstrated that a heterologous cell line, namely the mammalian HeLa cell line, may be successfully used to study the intracellular localization and trafficking of invertebrate virus proteins. The latter finding may be of significance for future studies of the intracellular trafficking of densovirus particles, especially in those cases where the corresponding host organism cell cultures are difficult to obtain or recalcitrant to laboratory conditions, or when carrying out experimental manipulation with the host cell culture is intractable for some other reason.

## 2. Materials and Methods

### 2.1. In Silico Analysis

To search for potential NLS motifs, cNLS Mapper [54] and Wolf PSORT [55] software were used. To search for NES motifs, the NetNes 1.1 software [56] was used. The translated amino acid sequences of the complementary DNAs (cDNAs) for capsid proteins, or open reading frames (ORFs) encoding the capsid proteins, for the densoviruses considered in this study were obtained from GenBank. The accession numbers were as follows: BgDV1 (AY189948), AalDV2 (X74945), AalDV3 (AY310877), AalDV1 (AY095351), HeDV (AY605055), AgDV (EU233812), AaeDV1 (M37899), AaeDV2 (FJ360744), CppDV (EF579756), BmDV (AY033435), CeDV (AF375296), DpDV (AY665654), HaDV2 (HQ613271), PpDV (JX110122), SfDV (JX020762), DpplDV (KF963252), MlDV (AY461507), CpDV (FJ810126), DsDV (AF036333), GmDV (L32896), JcDV (S47266), HaDV1 (JQ894784), PiDV (JX645046), PfDV (AF192260), BgDV2 (JQ320376), PcDV (AY032882), AdDV (HQ827781), AdMADV (KF275669), MpDV (AY148187), PmoHDV1 (DQ002873), PmoHDV3 (EU588991), PmoHDV2 (EU247528), PmoHDV4 (FJ410797), PmeDV (DQ458781), FchDV (JN082231), PchDV (AY008257), PmoPDV1 (GQ411199), PstDV2 (GQ475529), PstDV1 (AF273215), PmoPDV2 (AY124937), and IHHNV (AF218266). All amino acid positions for NLS and NES motifs in the text are given as they are in the translated amino acid sequences in the corresponding GenBank entries for each analyzed densovirus.

### 2.2. Plasmid Construction

The nucleotide sequences encoding BgDV1 capsid proteins VP1, VP2, and VP3 were amplified using the previously obtained pGEM-T-vector-based constructs containing the cDNAs for the corresponding BgDV1 proteins [51] as templates. The primers flanking the BgDV1 VP1-, VP2-, and VP3-coding sequences were designed and annealing temperature was calculated using the PrimerBlast software [57] available at http://www.ncbi.nlm.nih.gov/tools/primer-blast/. Amplification was performed in a Primus thermo cycler (MWG-Biotech, Ebersberg, Germany). Amplification conditions were as follows: initial denaturation 5 min at 95 °C, 30 cycles consisting of denaturation 1 min at 94 °C, primer annealing 1 min at the specific temperature depending on the primer pair used, and primer extension 3 min at 72 °C, final extension 7 min at 72 °C. The amplified DNA fragments were gel-purified and cloned into the pcDNA3.1/NT-GFP-TOPO vector (Invitrogen, Carlsbad, CA, USA) according to the manufacturer’s recommendations. Notably, this vector contains the SV-40 replication origin facilitating multicopy episomal replication of the vector in eukaryotic cell lines stably expressing the SV40 T-antigen (for example, in Cos-1 cell line), and neomycin resistance gene, encoding aminoglycoside phosphotransferase, which allows the selection of transfected cells using Geneticin (G-418). The accuracy of vector construction and the absence of PCR-introduced errors in the plasmid sequences were verified by Sanger sequencing. The obtained plasmid vectors containing the coding sequences for BgDV1 capsid proteins were purified and used for the transfection of mammalian cells.

### 2.3. Site-Directed Mutagenesis

Site-directed mutagenesis was performed following a previously described technique [58] with slight modifications. Namely, to introduce a nucleotide substitution into the extended DNA fragments corresponding to VP2 and VP3 coding sequences, a two-step PCR was carried out. The first step included the amplification of two DNA fragments overlapping by 25 nucleotides, with the region of overlap corresponding to the DNA region into which the mutation should be introduced. The reverse primer for the upstream fragment and the forward primer for the downstream fragment used in the first step contained the desired nucleotide substitution and the other two primers corresponded to the ends of the entire DNA fragment being mutated. In the second step, the extended DNA fragment was amplified using the overlapping DNA fragments obtained at the previous step as a template and the pair of primers flanking the entire mutated DNA fragment. Tersus Plus PCR kit (Evrogen, Moscow, Russia), containing a mixture of high fidelity polymerases, was used to perform PCR. The obtained extended DNA fragments were cloned into the pcDNA3.1/NT-GFP-TOPO vector (Invitrogen, USA) as described above. The mutagenesis and vector construction accuracy, as well as the absence of PCR-introduced errors, were all verified by Sanger sequencing.

### 2.4. In Vitro Transcription and Translation

The TnT^®^ Quick Coupled Transcription/Translation Systems (Promega, Madison, WI, USA) was used to perform verification/characterization of cloned genes (native and mutated). All procedures were performed according to the manufacturer’s recommendations. The obtained products were analyzed by protein electrophoresis and Western blotting.

### 2.5. Protein Electrophoresis and Western Blotting

Protein electrophoresis was performed in 10% sodium dodecyl sulfate-polyacrylamide gel electrophoresis (SDS-PAGE) according to the standard Laemmli technique [59]. Following electrophoresis, the proteins were transferred onto Amersham Hybond-LFP polyvinylidene difluoride (PVDF) membrane (GE Healthcare Life Sciences, Buckinghamshire, UK) by semi-dry electroblotting. Detection of BgDV1 VP proteins was performed using the ProtoBlot II AP System with Stabilized Substrate (Rabbit) (Promega, USA) according to standard procedures. Membranes were blocked with 5% bovine serum albumin (BSA) in Tris buffer saline with Tween-20 (TBST) (20 mM Tris-HCl, pH 7.5, 150 mM NaCl, 0.05% Tween-20). The previously described rabbit antibodies recognizing the epitope in the common C-terminal of BgDV1 capsid proteins [51] were used as the primary antibodies and the alkaline phosphatase-conjugated anti-rabbit antibodies supplied with the kit were used as the secondary antibodies. The protein bands of interest were revealed by adding the nitro-blue tetrazolium and 5-bromo-4-chloro-3'-indolyphosphate (NBT/BCIP)-containing substrate.

### 2.6. Cell Culture

The intracellular localization of BgDV1 capsid proteins was performed using the HeLa cell line. In certain experiments, the African green monkey kidney fibroblast-like cell line (Cos-1), suitable for transfection with the vectors requiring the expression of the SV40 T-antigen, was used. Cells were grown in flasks in Dulbecco’s modified Eagle medium (DMEM) medium supplemented with 10% bovine serum (Gibco, Gaithersburg, MD, USA) at 37 °C in 5% CO_2_ atmosphere. Cells were grown till they covered 70–80% of the growing area and were passaged approximately every five days. Cells were removed from the culture surface using trypsin/EDTA solution (PanEco, Moscow, Russia). Prior to transfection, cells were counted and then transferred to the Petri dishes containing culture medium to a final cell density of 1 × 10^5^. The bottom of the Petri dishes was laid with cover slips.

### 2.7. Transfection

The plasmid DNA used for transfection was purified with the Qiagen-tip 100 Plasmid Midi Kit (Qiagen, Germantown, MD, USA). Transfection was performed using Metafectene Pro (Biontex, San Diego, CA, USA) according to the manufacturer’s instructions. Namely, 5 μL of Metafectene Pro was mixed with 45 μL of 1× phosphate-buffered saline (PBS), and the necessary amount of plasmid DNA was adjusted with 1× PBS to a final volume of 50 μL in a separate tube. The two solutions were combined and incubated at room temperature for 15 min. After incubation, the mixture was added to the cells, and cells were incubated under normal growth conditions for 24 h. Then, the transfection mixture was replaced with fresh medium and cells were left to grow. After 24, 48, and 72 h of growth, cover clips with attached cells were taken from the Petri dishes and washed twice from the growth medium with 1× PBS before proceeding to fixing and immunostaining.

### 2.8. Immunohistochemistry

HeLa cells attached in a monolayer to cover slips were fixed with 4% paraformaldehyde (PFA) for 10 h at 4 °C. Then cover slips with cells were incubated with 0.1% Triton X-100 solution in 1× PBS for 7 min to improve antibody penetration. To block nonspecific antibody binding, cells were further incubated with blocking solution containing 4% BSA in 1× PBS for 24 hr at 4 °C. The previously described rabbit antibodies recognizing the epitope in the common C-terminal of BgDV1 capsid proteins or rabbit antibodies against GFP (Abcam, Cambridge, MA, USA), both at 1:1000 dilution, were used as the primary antibodies. The incubation with primary antibodies was performed for 2 hr with subsequent washing with 1× PBS. The FITC-conjugated anti-rabbit antibodies (Abcam, USA) were used as the secondary antibodies. Secondary antibodies were diluted 1:5000, and incubation was performed also for 2 hr with subsequent washing with PBS. The slides were mounted with ProLong Antifade Reagent (Invitrogen, USA) containing DAPI, and the cell nuclei were allowed to stain for several hours in the dark. The intracellular localization of the fluorescent signals was analyzed in a Carl Zeiss LSM 510 Meta confocal microscope.

## 3. Results and Discussion

### 3.1. BgDV1 Nuclear Transport Signal Prediction

**Figure 1:**
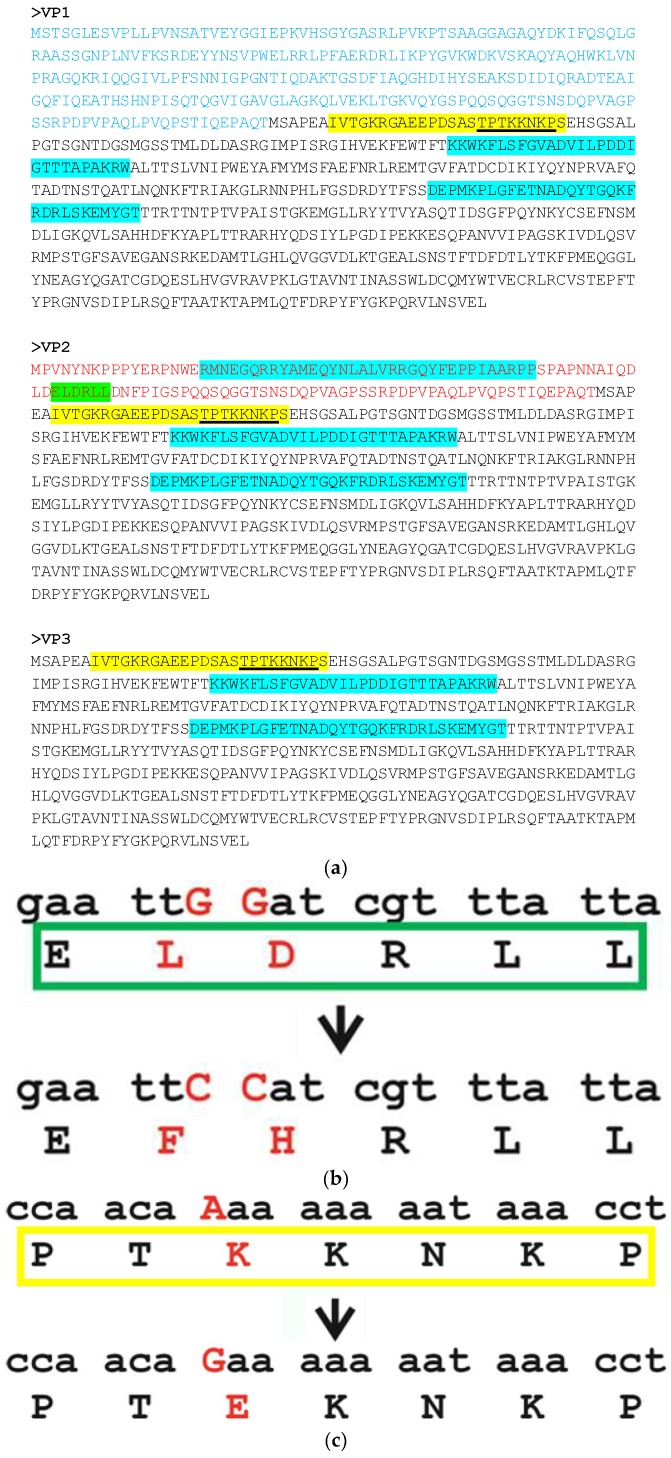
Schematic representation of the intracellular trafficking signals of *Blattella germanica* Densovirus 1 (BgDV1) capsid proteins: nuclear localization signal—NLS and nuclear export signal—NES. (**a**) Amino acid sequences of the native VP1, VP2, and VP3 proteins. The common C-end part of all BgDV1 capsid proteins corresponding to the entire VP3 protein is indicated with black font; unique N-end part of VP1 and VP2 are indicated with blue and red fonts, respectively. Underlining indicates the high-probability monopartite NLS. High- and low-score bipartite NLSs are highlighted by yellow and cyan backgrounds, respectively. A bright green background indicates the predicted NES in the unique N-end of VP2. Site-directed mutagenesis of the high-probability NLS in VP3 (**b**) and NES in VP2 (**c**). The nucleotides before and after the substitutions and the amino acids encoded by the corresponding triplets are highlighted by red upper-case letters. The original NLS is highlighted by the yellow box; the original NES—by the green box.

We demonstrated earlier that the German cockroach densovirus BgDV1 possesses three capsid proteins (VP1-VP3) with VP1 (97 kDa) and VP2 (80/85 kDa) proteins sharing a common C-terminal corresponding to the entire VP3 protein (57 kDa) and differing by their unique N-terminals [51] (Figure 1a). We used the Kosugi cNLS Mapper software and Wolf PSORT software (GenScript, Piscataway, NJ, USA) based on different algorithms for NLS prediction to look for potential NLSs in the amino acid sequences of the three BgDV1 capsid proteins. A single NLS monopartite motif (Pat7-like motif) was found by Wolf PSORT in the common C-end region of BgDV1 capsid proteins TPT**KK**N**K**P (here and in what follows, basic residues in NLS are underlined in bold) at amino acid positions 23–29 in the VP3 protein (respective amino acid (aa) positions 141–147 in the VP2 and 291–297 in VP1) (Figure 1, underlined on yellow background). This motif resembles the classical monopartite NLS with the consensus sequence K(K/R)X(K/R) (X—any amino acid residue) found in the c-Myc [22]. This motif in the common C-terminal of BgDV1 VP proteins was also found by the cNLS Mapper, but as a part of a high-score bipartite NLS (probability score 8.6) IVTG**KR**GAEEPDSASTPT**KK**N**K**PS at positions 7–30 in the VP3 (respective aa positions 125–148 in VP2 and 275–298 in VP1) (Figure 1, yellow background). This motif also corresponds well with the putative consensus sequence (K/R)(K/R)X_10–12_(K/R)_3/5_, where X is any amino acid residue and (K/R)_3/5_ indicates three out of five consecutive basic amino acids, suggested by Kosugi et al. [22]. In addition, a number of low-score bipartite NLSs were also predicted in both the unique N-terminals of VP1 and VP2 proteins and the common C-terminal. Among the NLSs with the highest scores were **KK**W**K**FLSFGVADVILPDDIGTTTAPA**KR**WA at amino acid positions 80–109 and DEPM**K**PLGFETNADQYTGQ**K**F**R**D**R**LS**K**EMYGT at amino acid positions 200–231 in the VP3 protein (respective aa positions 348–377 and 468–499 in VP1, and 198–226 and 318–349 in VP2), with a score of 3.0, and an **R**MNEGQ**RR**YAMEQYNLALV**RR**GQYFEPPIAA**R**PP motif with a score of 3.5 at the unique N-end of the VP2 protein (positions 18–51) (Figure 1, cyan background). The cNLS Mapper scores, using a β-glucuronidase (GUS)-GFP reporter protein fused to an NLS, were as follows: a GUS-GFP reporter protein fused to an NLS with a score of 8, 9, or 10 is exclusively localized to the nucleus, that with a score of 7 or 8 partially localized to the nucleus, that with a score of 3, 4, or 5 localized to both the nucleus and the cytoplasm, and that with a score of 1 or 2 are localized to the cytoplasm.

Using the NetNes 1.1 software, a canonical NES ELDRLL (here and in what follows, leucine residues in NES motifs are underlined) was predicted in the unique N-terminal of the VP2 protein (Figure 1, bright green background).

### 3.2. Nuclear Transport Signal Prediction for Other Densovirus Species

Although nuclear transport signals seem to play a pivotal role in the densovirus life cycle, only a limited number of works has been reported so far concerning the study of nuclear localization signals in densoviruses, and no data are available so far on the presence of NES in any of the known densoviruses. Therefore, we performed an in silico search for potential nuclear transport signals in the amino acid sequences of capsid proteins in the densoviruses not previously studied in this respect. Following the approach utilized for BgDV1, capsid protein sequences of the densoviruses belonging to five genera in the subfamily Densovirinae were analyzed using the cNLS Mapper and NetNES software. In this analysis, when the expression strategy has been described for the virus and cDNAs encoding capsid proteins are known, we used the corresponding translated amino acid sequences for the capsid proteins available in GenBank; when only the genome structure has been revealed for a particular densovirus, we used the translated amino acid sequences for the ORFs encoding capsid proteins. We considered significant the NLS motifs predicted by cNLS Mapper with a score of 6 or higher, since according to Kosugi et al. [54], the NLSs predicted with a score higher than the indicated threshold were able, when fused to the reporter protein, to localize it, exclusively or partially, to the nucleus. In certain cases, we also used the Wolf PSORT software to gain further support for the predicted NLS motifs.

We first considered the NLS motifs in the densoviruses from the four Densovirinae genera as far as nuclear localization signals in the genera *Penstyldensovirus* and *Hepandensovirus* have already been extensively studied earlier [27], and further proceeded with the analysis of NESs in all five Densovirinae genera.

The group of closely-related lepidopteran densoviruses GmDV, MlDV, JcDV, HaDV1, PiDV, and DsDV revealed the presence of a bipartite NLS motif with the consensus sequence ExT**KRK**A(D/G)(S/T)(S/P)(A/V)xE(T/G)P(A/S)**KK**G(A/T)(T/H), where “x” is any amino acid. The cNLS Mapper score for this motif ranged from 6 to 7. The motif was found in a unique part of the VP3 protein, and thus was shared by the VP1, VP2, and VP3 proteins, but not the VP4 protein. It should be noted that according to the expression strategy interpreted for this group of viruses, all four capsid proteins VP1–VP4 are encoded by a single transcript and are translated from the first four AUG codons in frame so that all four proteins contain the VP4 sequence as a common C-terminus, while the VP1–VP3 proteins possess unique N-terminal parts [1,3,5,7]. The similarity of the predicted NLS motifs is quite expected given the high homology of VP proteins between these representatives of the genus *Ambidensovirus* and the fact that their insect hosts belong to the same Lepidoptera order. It should be noted that the presence of a basic NLS motif in the unique region of VP1 was previously reported for JcDV [31].

In the single ORF encoding capsid protein(s) of CpDV [10], we found a lysine-rich motif LN**K**TGYGSNVSFTEFTAG**K**PMIPS**K**IA**K**P with a score of 7.3. Although the expression strategy for CpDV has been discussed already, there is not clear data on how capsid proteins are synthesized during the virus life cycle. However, it was suggested that the three capsid proteins of CpDV may be translated from the three successive AUG codons, as is the case with other *Densovirus* genus representatives [10]. If so, then the detected NLS motif is located in the common C-terminal part of all VPs.

In PfDV, the larger VP protein encoded by ORF 6, we found a putative monopartite NLS GEPPN**KK**A**K**TG (score 8.0), which resembles the classical c-myc NLS, and is somewhat similar to the TPT**KK**N**K**P motif found in the BgDV1 capsid proteins. No NLS motifs were predicted in the PfDV smaller VP protein (ORF 5).

Interestingly, both cNLS Mapper and Wolf PSORT predicted no significant NLS motifs in any capsid proteins of AdDV, although a number of low-score bipartite motifs were predicted by cNLS Mapper, with the D**K**G**K**G**KR**GGGG**R**PP**K**SSGG**KR**S**R** motif (score 4.3) being shared by VP1, VP2, and VP3 capsid proteins, resembling the case with BgDV1 capsid proteins.

In the capsid protein of *Acheta domesticus* mini ambidensovirus (AdMADV), we predicted a high-score monopartite NLS motif N**R**L**KR**L**R**LAAE (score 8.0).

We also analyzed the translated amino acid sequences of the ORF1 and ORF2 encoding the capsid proteins of BgDV2 (BgDV1-like densovirus). A single monopartite NLS TYR**KK**W**R**FL of the c-myc type was predicted in the larger ORF1 (score 6.5), which, however, appeared to be rather different in its amino acid composition from the one found in BgDV1.

Analysis of the translated ORFs encoding the capsid proteins of PcDV showed that the smaller VP coding ORF contained a high-score (9.5) putative monopartite P**R**VS**KK**P**R**IS NLS, which may also form a part of a bipartite NLS WY**RK**YQFVNNLND**K**GQP**R**VS**KK**P**R**IS (score 6.3).

In the translated amino acid sequence of the smaller ORF encoding MpDV capsid proteins, a putative monopartite NLS motif NE**RKR**I**K**LG was predicted by cNLS Mapper (score 7). Another NLS was found in the larger VP coding ORF—the bipartite S**KR**PADSSGSEPAP**KR**AGGT motif was predicted with a high score of 9.0.

All representatives of the genus *Iteradensovirus*, namely BmDV, CeDV, DpDV, PpDV, SfDV, and DpplDV, showed the presence of the putative bipartite NLS in the N-terminal parts of their translated amino acid sequences of the VP coding ORF, although with a rather low score of 6. This motif, with the consensus sequence of GMG**KRK**(S/N)(T/N)E**K**DWA(**K**/T)I**KR**IN(**R**/T), was highly conserved in five densoviruses, namely BmDV, CeDV, DpDV, PpDV, and DpplDV. In SfDV, the NLS motif contained a longer stretch of amino acid residues (PNEDNI) instead of the (S/N)(T/N) consensus between the conserved **KRK** and E**K**D regions. Since CeDV, as well as BmDV, and apparently all other iteradensoviruses, possess five/four capsid proteins which are most probably translated from the successive in-frame ATGs [17,60], the predicted NLS motif should be localized in the unique N-terminal region of the VP2 protein and hence be shared by the VP1 and VP2 proteins. Wolf PSORT search confirmed the presence of the conserved bipartite NLS in all six members of the genus *Iteradensovirus*. In addition, using this software we found a conserved monopartite NLS PVT**R**S**KK** with the consensus sequence of the c-myc type close to the C-end of the VP protein coding ORF in four densoviruses, namely CeDV, PpDV, SfDV, and DpplDV.

Surprisingly, our analysis predicted no NLSs with significant score in the amino acid sequence of HaDV2, another representative of the genus *Iteravirus*, although we found a **K**NFTEE**K**IVG**R**PLYGMPT**K**DWG**R**I**KK**I sequence (score 2.9) somewhat resembling the putative bipartite NLS motif almost at the same amino acid position in the VP, as in other members of *Iteravirus*.

All the analyzed mosquito-infecting densoviruses belonging to the genus *Brevidensovirus* revealed the presence of a monopartite SV40 T-antigen-like NLS in the closest proximity of the start methionine of their VP1/VP2 protein with the highest possible score of 10–11. Three densoviruses, AalDV3, HeDV, and AalDV2, which were shown to be closely related based on their whole genome sequences [61], contained the same **R**GT**KRKR**EAD sequence located close to the N-terminus of the ORF encoding capsid proteins in the presumable smaller VP2 capsid protein. This motif may therefore be shared by both VP1 and VP2 proteins. The NLS motif in the other densoviruses from this genus contained the **R**GT**KRKR** sequence in common with the first *Brevidensovirus* group with variations in the remaining three amino acid residues (namely **R**GT**KRKR**ETG in AalDV1, **R**GT**KRKR**DAG in AaeDV1, **R**GT**KRKR**GAG in AaeDV2, and the same **R**GT**KRKR**GAE motif in AgDV and CppDV).

The presence of potential NLSs similar to that of the SV40 large T-antigen was shown previously for the densoviruses from *Aedes aegypti* and *Aedes albopictus*. The motifs were localized at the very N-end of the VP proteins [39,40]. Li et al. [13] studied the roles of N- and C-terminal sequences of capsid proteins in the *Aedes albopictus* C3/36 cell densovirus (AalDV1 according to current taxonomy) capsid formation, and showed that although the predicted NLS motif GT**KRKR** shared by both VP1 and VP2 was not essential for virus particle assembly, it affected the formation of crystalline arrays in the infected Sf9 cells.

The comprehensive in silico analysis of the putative NLS motifs in both regulatory and capsid proteins of the penaeid densoviruses belonging to the group of PstDV-related small-genome densoviruses (genus *Penstyldensovirus*) and PmergDV-related densoviruses with large genomes (genus *Hepandensovirus*) was performed earlier by Owens et al. [27]. In the translated sequences of the ORF3 encoding the capsid protein VP1 of the densoviruses from *P. monodon* and *P. merguiensis*, a very strong NLS (P**KKKKK**Y**K**) was predicted, closely resembling the NLS of SV40 T-antigen (PKKKRKV). It was observed that in the densovirus from *P. chinensis*, the leading proline (P) was replaced with a glutamine (Q) leading to the Q**KKKKK**Y**K** motif. It should be noted that all the predicted NLS motifs localized close to the C-end of the capsid protein. In the PstDV-related densoviruses, the capsid protein VP1 was shown to have only one possible monopartite NLS (**PRKR**S**RRD**) located in ORF3. This signal also resembles the SV40 T-antigen NLS. However, it was regarded by the authors as most probably nonfunctional due to the presence of potentially disruptive S and D residues, and this supposition was supported by experimental evidence.

Our findings indicate that apparently all the analyzed densoviruses belonging to different genera contain one, or sometimes two, potential nuclear localization signals. Only for AdDV and HaDV2 the NLSs were predicted with rather low scores. Considering only the densoviruses with known expression strategies of their genomes, we can see that in most cases, the predicted NLSs are not located exclusively in the unique N-terminal part of the largest VP, but are shared by all VPs, sometimes with the exception of the smallest (VP3 and/or VP4) proteins. Interestingly, densoviruses whose hosts belong to the same taxonomic group have NLS motifs resembling each other in the structure and position in the capsid protein, although the correlation is not absolute.

In the case of nuclear export signals, the situation appeared to be somewhat different from that observed with NLSs. Surprisingly enough, the NetNES software was not able to predict the presence of any conventional NES motifs containing hydrophobic amino acid residues in members of the lepidopteran ambidensoviruses. At the same time, other representatives of the genus revealed the presence of putative NES sequences in their VP protein coding ORFs. Namely, a high-probability leucine-rich NES motif LEKEKFDLETL was predicted at the aa position 97–107 in the single CpDV VP-coding ORF. We also found a stretch of amino acid residues IELPISVDDDVHI at the aa position 67–79 in the PfDV smaller capsid protein coding ORF (ORF5), potentially containing a nuclear export signal. A putative NES motif LTLRDFSL was predicted close to the first methionine of the largest AdDV VP1 protein in its unique N-terminal region (aa position 8–15), but no NES motifs could still be predicted in AdMADV. As for the BgDV2, a rather strong putative IDNFDLDLL NES motif was found in its larger VP ORF1 (at aa position 59–67), while a less probable NES was also predicted in the smaller VP ORF2 the aa positions 286–299. It should be noted that the NES motif in the larger VP ORF is located almost at the same position as in the corresponding large VP coding ORF of BgDV1 which represents rather close similarity between the amino acid sequences of the two viruses. A stretch of amino acids, LDKKAIRGALQV, containing a possible NES motif, was found in the smaller MpDV VP coding ORF starting at aa position 89–100. As was the case with lepidopteran ambidensoviruses, no putative NES motifs were predicted in either of the two PcDV capsid protein ORFs.

Interestingly, among the representatives of the genus *Iteravirus*, only CeDV showed the presence of a putative NES within the LLMYLSEGEYLRL sequence, starting at the aa position 283–295 of its VP ORF.

Regarding representatives of the genus *Brevidensovirus*, the NetNES software predicted a highly probable NES motif of slightly varying amino acid composition in the VP1/VP2 capsid proteins in all brevidensoviruses except for AaeDV1, namely IMKALNRVAL in AgDV (at aa position 109–118 of the VP ORF), IMKALNKVAL in CppDV (at position 109–118), IMKALNNVSL in AalDV1 (at position 111–119), and IMKALNAVAL in a group consisting of AaeDV2 (at position 111-120), AalDV3 (at position 108–117), HeDV (at position 108–118), and AalDV2 (at position 108–118). For AgDV and CppDV, the amino acid residues possibly forming a part of a NES were found in the C-end part of the respective capsid proteins.

We performed also a search for potential NESs in the capsid protein coding ORFs of penaeid densoviruses. We were not successful in finding any significant NES motifs in representatives of the genus *Penstyldensovirus*, although certain amino acid residues showed a tendency to be a part of a potential NES. In contrast, all the analyzed hepandensoviruses showed the presence of the putative NES motif LLAIEIAL in their VP protein starting at aa position 174–181.

To summarize, unlike the case with NLS motifs, not all densoviruses showed the presence of canonical NES motifs in their VP proteins, with representatives of the genera *Iteravirus* (except for CeDV) and *Penstyldensovirus*, as well as the lepidopteran ambidensoviruses PcDV and AdMADV lacking them. It may be that the capsid proteins of these densoviruses contain noncanonical nuclear export signals, as described previously for the MVM [45]. We may also speculate that these viruses, or some of them, might not use the transport through the nuclear pore complex to exit the nucleus, but may instead induce nuclear membrane disruption, as do other non-enveloped viruses [42].

A summary of nuclear traffic signals which were predicted in five Densovirinae genera is presented in Table 1.

It was shown for human parvovirus B19 that the NLS is exposed on the surface of an isolated VP2 subunit and, thus, can be recognized by cellular nuclear import molecules. After assembly, the NLS is hidden because it is on the inner capsid surface [62]. It would be of particular interest to analyze the spatial organization of NLSs within the assembled capsid in densoviruses.

To date, using the X-ray diffraction method, the spatial structures of the capsids of four densoviruses, namely GmDV, BmDV, PstDV, and AdDV (PDB codes: 1DNV, 3P0S, 3N7X, and 4MGU, respectively), have been described [8,63,64,65] and the three-dimensional structures of capsids of AalDV2 and JcDV were determined by electron cryomicroscopy and computer reconstruction [6,66]. In these studies, both native virus capsids [8,63,66] and virus-like particles (VLP) formed during expression of certain capsid proteins and their combinations [6,64,65] were used.

Unfortunately, we were unable to locate the NLS regions of capsid proteins within the spatial structures using data presented in the literature [6,8,63,64,65,66]. Firstly, in studies that investigated the structure of the VLP, the capsid proteins containing no NLS (or containing NLS with extremely low score) were used to form these VLPs. It is assumed that densovirus capsid proteins without NLS enter the nucleus of the infected cell in a complex with other capsid proteins with functional NLS [42]. Secondly, in all other studies to date, NLS regions were not visible, suggesting that they are highly disordered e.g., [6].

### 3.3. Intracellular Localization of BgDV1 Capsid Proteins

We performed functional analysis of the in silico predicted nuclear transport signals in BgDV1. First, we amplified the previously obtained cDNA fragments encoding BgDV1 capsid proteins VP1, VP2, and VP3 [51] and cloned the obtained fragments into the pcDNA3.1/NT-GFP-TOPO mammalian expression vector (Invitrogen, USA). As a result we obtained three vector constructs, pTOPO_GFP_VP1, pTOPO_GFP_VP2, and pTOPO_GFP_VP3, which allowed the expression of BgDV1 capsid proteins N-fused with GFP in the mammalian cell line under the control of the cytomegalovirus (CMV) promoter. The accuracy of cloning and the absence of PCR-induced errors in the cloned sequenced were verified by sequencing the obtained constructs.

**Figure 2:**
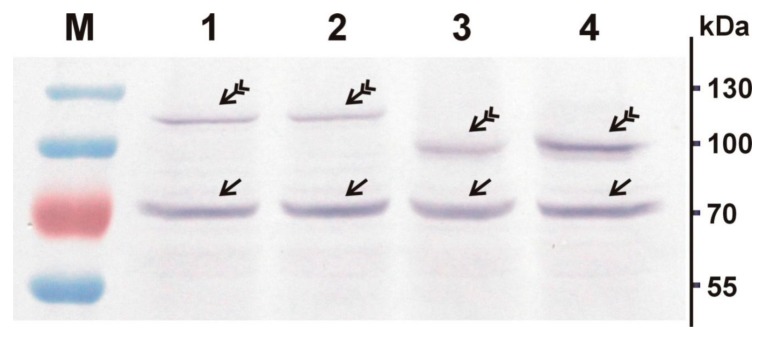
Western blot analysis of the in vitro translated native and mutant BgDV1 VP2 and VP3 capsid proteins N-fused to green fluorescent protein (GFP). pcDNA3.1/NT-GFP-TOPO (Thermo Fisher Scientific, Waltham, MA, USA) expression vector-base constructs containing the coding sequences for native VP2 and VP3 proteins (GFP-VP2 and GFP-VP3, respectively), and for the corresponding proteins with introduced mutations in NES and NLS (GFP-VP2mut and GFP-VP3mut, respectively) were in vitro translated using rabbit reticulocytes based TnT^®^ Quick Coupled Transcription/Translation Systems (Promega, USA). The in vitro translation products were analyzed by Western blotting using rabbit antibodies against the epitope in the common C-end parts of VP2-VP3 proteins (primary antibodies) and goat anti-rabbit secondary antibodies. Lane 1—GFP-VP2, lane 2—GFP-VP2mut, lane 3—GFP-VP3, lane 4—GFP-VP3mut. M—protein molecular weight marker. Feathered arrows indicate the corresponding GFP-fused BgDV1 capsid proteins. Plain arrows indicate the bands corresponding to rabbit immunoglobulin (Ig) heavy chain.

To test the performance of the obtained constructs, we also carried out in vitro translation using the coupled in vitro transcription/translation system based on rabbit reticulocyte lysate, and analyzed the obtained products using Western blot (Figure 2). First, the performance of the genetic constructs expressing the VP2 and VP3 proteins was analyzed in this system. In the case of the pTOPO_GFP_VP2 (Figure 2, lane 1) and pTOPO_GFP_VP3 (Figure 2, lane 3) constructs, we could clearly see the bands corresponding to the fusion proteins GFP-VP2 and GFP-VP3, respectively (indicated by feathered arrows) which imply that the obtained expression constructs are fully functional and densovirus proteins can be successfully produced in the mammalian system in sufficient quantities. Notably, in all the analyzed translation reaction samples, we also observed the presence of an additional ~70 kDa band (indicated by plain arrows in Figure 2), corresponding to rabbit immunoglobulin heavy chain.

Nevertheless, it should be noted that the observed molecular weights of the GFP-VP2 and GFP-VP3 fusion proteins, 118 kDa and 100 kDa, respectively, were higher than the corresponding predicted molecular weight, 98.7 kDa for GFP-VP2 and 85.5 kDa for GFP-VP3. We have previously observed similar discrepancies between the predicted and observed molecular weight for the native BgDV1 capsid proteins when resolved by SDS-PAGE [51]. These discrepancies may be attributed to the specific amino acid composition and/or secondary structure of BgDV1 proteins leading to altered mobility in the electric field, a phenomenon commonly discussed in the literature (e.g., [67,68]). The possibility of some post-translational modifications as the cause of the observed higher molecular weight should also be considered.

HeLa cells were transfected with the three vector constructs pTOPO_GFP_VP1, pTOPO_GFP_VP2, and pTOPO_GFP_VP3, encoding BgDV1 capsid proteins fused with the green fluorescent protein. Following transfection, we performed immunolocalization studies using two types of FITC-conjugated antibodies: antibodies recognizing the epitope in the common C-end part of the capsid proteins (C-end) and antibodies recognizing the epitope in the GFP moiety of the fusion protein (N-end). Cell nuclei were counterstained with DAPI. It should be noted that we failed to detect bright GFP signals, probably because of the properties of the optical filters in our fluorescence detecting system. Therefore, we used GFP-specific primary antibodies fused with FITC to visualize the localization of the GFP fusions, which allowed us to obtain a stable and much brighter signal.

**Figure 3:**
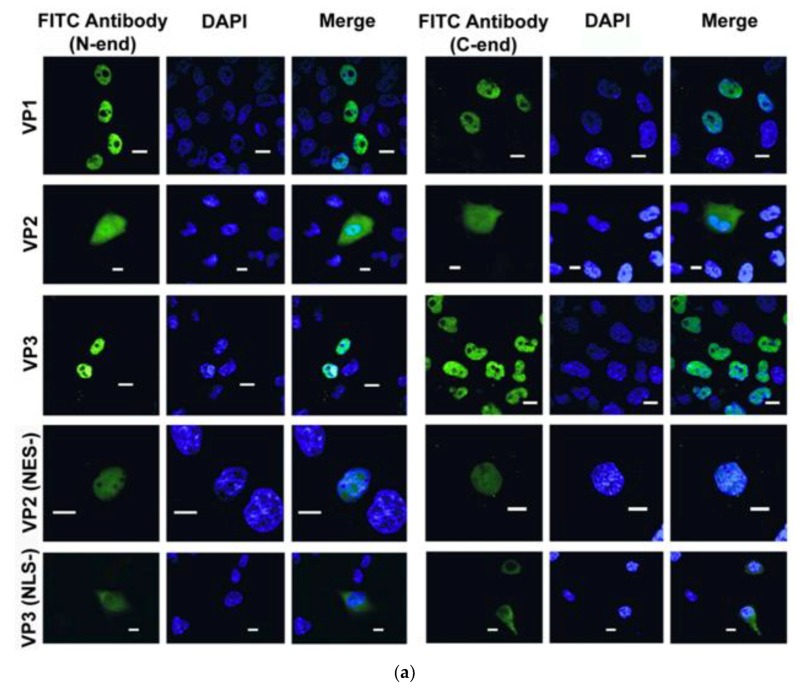

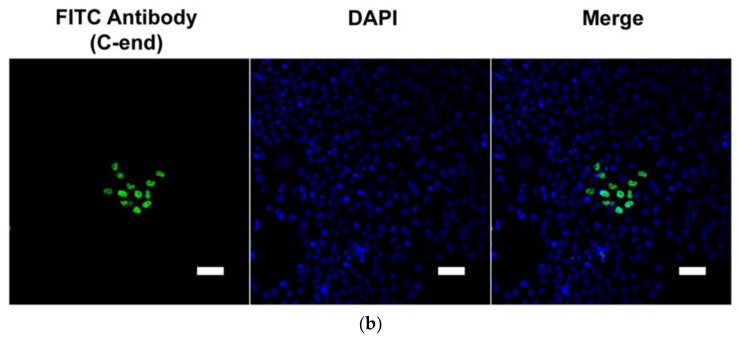
Indirect immunofluorescence of HeLa cells transiently expressing the capsid BgDV1 proteins fused with GFP. (**a**) The transfected cells transiently expressing N-fused with GFP native VP1 (VP1), VP2 (VP2), VP3 (VP3) proteins, or VP2 and VP3 proteins with mutated NES and NLS (VP2 (NES–) and VP3 (NLS–), respectively) were fixed in 4% PFA and subsequently immunostained with both primary rabbit antibodies against the GFP moiety (left panel), or against the common C-end part of BgDV1 capsid proteins (right panel), and goat anti-rabbit FITC-conjugated secondary antibody. The corresponding immunostaining results are presented in the left and right panels (FITC antibody (N-end) and FITC antibody (C-end), respectively). Cell nuclei were counterstained with DAPI. The intracellular localization of the fluorescent signals was analyzed by confocal microscopy. Scale bar—10 µm. (**b**) Panoramic view of the immunostained HeLa cells transiently expressing N-fused with GFP native VP1. Scale bar—50 µm. The viral protein is detected only in a small number of cells. Apparently, all cells expressing the foreign genetic material are descendants of one cell.

Immunostaining with both the N-end and C-end antibodies revealed that GFP-VP1 and GFP-VP3 fusion proteins accumulated in the nuclei of the transfected HeLa cells (Figure 3a, left and right panels, VP1 and VP3 rows). At the same time, GFP-VP2 protein localized both in the nuclei and in the cytoplasm, as demonstrated with both N-end (Figure 3a, left panel, VP2 row) and C-end (Figure 3a, right panel, VP2 row) antibodies.

As described above, the in silico analysis of the amino acid sequences of BgDV1 predicted a number of motifs potentially controlling the intracellular trafficking of capsid proteins. Among them are a number of NLSs with different probability scores in the common C-end of all capsid proteins and in the unique N-end of the VP2 protein and a NES also in the unique VP2 N-end (Figure 1a). We may thus infer that a single NLS or several NLSs direct the nuclear import of the virus capsid proteins, while NES may direct the export of the VP2 capsid protein, and probably also the mature progeny virus particle, from the nuclei of virus-infected cells. The immunolocalization study (Figure 3a) demonstrated that VP1 and VP3 proteins indeed accumulated in the nucleus, which corresponds well with the presence of NLS motifs. At the same time, VP2 protein was observed also in the cytoplasm, which suggests that the accumulation of this protein in both cellular compartments may be a result of the interplay between the two signals, NLS and NES.

While analyzing the intracellular location of BgDV1 capsid proteins, we made an extremely interesting observation. We found that the number of HeLa cells expressing the fusion proteins was quite low, with the protein-producing cells being localized in clusters, thus pointing to their possible clonal origin. A panoramic view of the immunostained HeLa cells transiently expressing VP1 protein N-fused with GFP is shown in Figure 3b. The same results were observed for all the analyzed constructs.

This phenomenon may result from the low efficiency of transfection of HeLa cells, when a single cell containing a plasmid (probably, integrated into cell genome) transfers it to its progeny. However, the transfection of HeLa cells by the control construct expressing GFP under the CMV promoter showed that the amount of transfected cells corresponds to the expected given the 75–80% transfection efficiency.

Another explanation may involve epigenetic regulation. To test this hypothesis, in the first series of experiments we used HeLa cells transfected with GFP_VP1, GFP_VP2, and GFP_VP3 constructs to obtain stable cell lines resistant to G-418. The expression vector pcDNA3.1/NT-GFP-TOPO contains a neomycin resistance gene, encoding aminoglycoside phosphotransferase, which allows the selection of transfected cells using G-418. If upon the transfection with the indicated constructs, the cells are placed into the medium containing G-418, only the cells producing the resistance protein, and consequently bearing the corresponding plasmid will be able to grow. In our case, we observed that HeLa cells grew to high numbers indicating that a high percentage of the cells successfully received the plasmid and produced aminoglycoside phosphotransferase, although they showed no expression of GFP-VP proteins. In other words, in the medium with G-418 the transfected cells express the protein necessary for antibiotic resistance which is encoded in the vector construct used for transfection, but they do not express the GFP-fused protein which is encoded in the other part of the same construct.

Next, we analyzed the expression of the GFP-VP3 fusion protein in the Cos-1 cell line stably expressing SV40 T-antigen. It is well-known that vectors containing the SV-40 origin of replication are able to replicate independently in Cos-1 cells and exist in the cell as multicopy episomes. It is noteworthy that the intracellular localization of the analyzed protein was the same as it was in the case of HeLa cell transfection. The intracellular localization of the GFP-VP3 protein during its transient expression in Cos-1 cells transfected with the corresponding expression construct is shown in Figure S1 (Supplemental Material). However, only individual cells revealed the expression of GFP-VP3 protein, similar to what we observed with HeLa cells. We also obtained the Cos-1 cell line stably resistant to G-418. In this case, similar to what was described above, the ability of cells to grow in medium containing G-418 means that the cells express aminoglycoside phosphotransferase; however, no expression of GFP-VP3 protein was observed again. We believe that these results may be interpreted as evidence that epigenetic regulation might operate in the transfected cells.

It may be assumed that the expression of GFP-fused BgDV1 capsid proteins in the mammalian cells may be suppressed by the activity of epigenetic mechanisms due to some intrinsic cytotoxic properties of the virus protein, and this suppressed state may be transmitted through generations of cells. Interestingly, it has been demonstrated previously by Liu et al. [69] that the expression of a number of modified GFP forms, namely, the enhanced GFP and red-shifted GFP, resulted in the cells expressing these proteins undergoing apoptosis. However, in a number of cells in the population the repression mechanisms might not have been activated for some reason, leading to them producing the GFP-fusion proteins and passing this property to their daughter cells. It should be noted that due to the low number of cells producing the BgDV1-GFP fusion proteins, we failed to obtain a visible band for any of the proteins using Western blot.

The main challenge in studying the intracellular trafficking of both the densovirus capsid proteins and virus particles in many cases is the absence of a suitable model system [31,60]. It is quite reasonable that the most adequate model system in the case of each densovirus would be cell lines obtained from the corresponding insect host. However, in actual practice it is often impossible to obtain a stable cell line for particular insects, or cell lines may not be easily maintained in the laboratory or may not be suitable for certain molecular genetic manipulations, for example, cell transfection. As we demonstrated here, a tractable solution is to use a heterologous cell culture which is easy to manipulate and maintain under laboratory conditions, and which is widely used to carry out molecular biology studies; many mammalian cell lines may serve this purpose. However, it must be experimentally demonstrated that the mammalian cell line-based model system is functional for each particular case, especially because mammals and insects are separated by millions of years of evolution. On the other hand, however, nuclear localization and nuclear export signals are known to be highly evolutionarily conserved in lower through higher eukaryotes.

From our findings, we can conclude that, notwithstanding the relatively low number of cells expressing the target proteins, the mammalian cell line may serve as an adequate and tractable model system to analyze the intracellular localization of capsid proteins of the invertebrate parvovirus.

### 3.4. Functional Analysis of the Nuclear Localization and Nuclear Export Signals in BgDV1 Capsid Proteins

To provide further experimental support for the hypothesis that in silico-predicted nuclear trafficking signals are functional, and to directly test the possible functional activity of the predicted NLS and NES motifs, we mutated the putative cellular trafficking signals to inactivate them and then analyzed the intracellular localization of the corresponding capsid proteins.

To test the functional activity of the nuclear import signals, we chose the NLS motif TPT**KK**N**K**P in the common C-end part of BgDV1 capsid protein as the motif with the highest probability score, which should have the highest probability to be functionally active. Using site-directed mutagenesis, we introduced an A → G mutation in this motif, which led to the substitution of the first lysine in the NLS motif in the VP3 capsid protein with glutamic acid (K → E) (Figure 1b). To test the functional activity of the predicted NES motif in the VP2 protein, we changed two GG nucleotides to CC nucleotides (GG → CC) which resulted in two amino acid substitutions (LD → FH) (Figure 1c). The resulting constructs were designated pTOPO-GFP-VP2mut and pTOPO-GFP-VP3mut.

It should be noted that the search for potential NLSs in the amino acid sequence of VP3 using both the Wolf PSORT and cNLS Mapper software revealed only the presence of the previously described low-score motifs (Figure 1a) and not the high-score TPT**KK**N**K**P motif. Similarly, using the NetNes software we were not able to predict the presence of NES in the VP2 protein containing the two described mutations.

The accuracy of site-directed mutagenesis was verified by Sanger sequencing. As was the case with the previously described expression constructs, to test the performance of the TOPO-GFP-VP2mut and TOPO-GFP-VP3mut expression constructs, we carried out in vitro translation using the coupled in vitro transcription/translation system and analyzed the products using Western blot (Figure 2). In the case of the pTOPO_GFP_VP2mut (Figure 2, lane 2) and pTOPO_GFP_VP3mut (Figure 2, lane 4) constructs, we could see the bands corresponding to the fusion proteins GFP-VP2mut and GFP-VP3mut, respectively (indicated by feathered arrows). It was also evident that GFP-VP2mut and GFP-VP3mut proteins have similar molecular weights to the native GFP-VP2 and GFP-VP3 fusion proteins, which indicated that the introduced mutations did not lead to the appearance of any stop codons and the synthesis of truncated protein forms, and that the corresponding protein pairs apparently differed only by the introduced amino acid substitutions. Hence, both constructs were fully functional in the mammalian system and could be used for the transfection of HeLa cells.

Immunostaining studies using the C-end antibodies revealed that the intracellular localization of the GFP-VP2mut protein with mutated NES changed compared to the intracellular localization of the intact protein. The mutated protein was characterized predominantly by nuclear localization (Figure 3, right panel, VP2 (NES–) row), which most probably resulted from the activity of the intact NLSs. At the same time, almost no protein could be observed in the cytoplasm, implying that NES indeed leads VP2 protein transport into the cytoplasm. This finding is thus the first demonstration of the presence of a functionally active canonical NES in the densovirus capsid proteins.

The study of the intracellular localization of the GFP-VP3mut protein with inactivated NLS showed that the mutated protein accumulated both in the cytoplasm and the nuclei of transfected cells (Figure 3, right panel, VP3 (NLS–) row), and therefore changed its subcellular localization compared to the native protein. Immunostaining using antibodies against the GFP moiety (N-end antibodies) showed the same results as with the C-end antibodies for both the GFP-VP2mut and GFP-VP3mut proteins (Figure 3, left panel, VP2 (NES–) row and VP3 (NLS–) row, respectively).

In the case of the GFP-VP3mut protein lacking the high-score NLS motif, we observed that a small portion of the protein still accumulated in the nucleus (Figure 3, left and right panels, VP3 (NLS–) row). This observation may be accounted for by the mutated NLS partially retaining its activity, or that one or several low-score bipartite NLS motifs are also able to direct the nuclear import of the BgDV1 capsid protein, but only at a very low level; the latter possibility seems to us to be more probable.

We conclude that both the nuclear export signal in VP2 and nuclear localization signal in VP3, and hence in other BgDV1 capsid proteins, are fully functional and may control the transport of the newly synthesized BgDV1 capsid proteins to the nucleus for subsequent virus capsid assembly, and apparently, the exit of the mature virus capsids from the nucleus to the cytoplasm of the virus-infected cells.

## Figures and Tables

**Table 1:** Summary of nuclear traffic signals that were predicted in five Densovirinae genera in the present work.

Genus	Virus	NLS *	Score	VP Protein (VP-Coding ORF) Containing NLS Motif **	NLS Amino Acid Positions ***	NES	VP Protein (VP Coding ORF) Cjntaining NES Motif **	NES Amino Acid Positions ***
*Ambidensovirus*	Lepidopteran densoviruses	Consensus motif:ExTKRKA(D/G)(S/T)(S/P)(A/V)xE(T/G)P(A/S)KKG(A/T)(T/H), “x” is any amino acid		VP1	326–346	No		
				VP2	50–70			
				VP3	3–23			
	GmDV	EATKRKADSPAVETPAKKGTT	6.0					
	MlDV	EPTKRKAGSSAAETPAKKGAT	6.5					
	JcDV	EGTKRKADTPVEEGPSKKGAH	6.5					
	HaDV1	EATKRKADTPAEEGPSKKGAH	7.0					
	PiDV	EGTKRKADSPVEEGPSKKGAH	6.5					
	DsDV	ESTKRKADTPAEETPSKKGAH	7.5					
	CpDV	LNKTGYGSNVSFTEFTAGKPMIPSKIAKP	7.3	VP ORF1	399–427	Yes	VP ORF1	97–107
	PfDV	GEPPNKKAKTG	8.0	VP ORF6	126–136	Yes	VP ORF5	67–79
	AdDV	DKGKGKRGGGGRPPKSSGGKRSR (low score)	4.3	VP1	365–387	Yes	VP1	8–15
				VP2	146–168			
				VP3	16–138			
	BgDV1	TPTKKNKP(probably part of IVTGKRGAEEPDSASTPTKKNKPS)	8.6 ^#^	VP1	291–297(275–298)	Yes		
				VP2	141–147(125–148)		VP2	64–69
				VP3	23–29(7–30)			
	BgDV2	TYRKKWRFL	6.5	VP ORF1	182–190	Yes	VP ORF1	59–66
							VP ORF2	286–299
	PcDV	PRVSKKPRIS(probably part of WYRKYQFVNNLNDKGQPRVSKKPRIS)	9.5(6.3)	smaller VP coding ORF	74–83(58–83)	No		
	MpDV	NERKRIKLG	7.0	smaller VP coding ORF	16–24	Yes	smaller VP coding ORF	89–100
		SKRPADSSGSEPAPKRAGGT	9.0	larger VP coding ORF	105–124			
Unassigned	AdMADV	NRLKRLRLAAE	8.0	VP (ORF VP1)	127–137	No		
*Iteradensovirus*	BmDV	GMGKRKSTEKDWAKIKRINR	6.5	N-terminal part of VP ORF (most probably VP1 and VP2 proteins)	94–113	Only CeDV		
	CeDV	GMGKRKSTEKDWAKIKRINT	6.0		94–113		VP ORF (most probably all VPs)	283–295
	DpDV	GMGKRKNTEKDWATIKRINR	5.5		92–111			
	PpDV	GMGKRKSNEKDWATIKRINR	6.0		94–113			
	DpplDV	GMGKRKSTEKDWAKIKRINR	6.0		94–113			
	SfDV	GMGKRKPNEDNIEKDWAKIKRINR	6.0		94–117			
	CeDV	PVTRSKK	-- ^##^	C-terminus of VP ORF (most probably common C-terminus of all capsid proteins)	668–674			
	PpDV	PVTRSKK	--		663–669			
	SfDV	PVTRSKK	--		672–678			
	DpplDV	PVTRSKK	--		667–673			
	HaDV2	KNFTEEKIVGRPLYGMPTKDWGRIKKI (low score)	2.9	N-terminal part of VP ORF (VP1 and VP2)	79–105	No		
*Brevidensovirus*		Consensus motif:RGTKRKRx(A/T)x		N-terminus of VP ORF (most probably VP1 and VP2)				
	AalDV3, HeDV, AalDV2	RGTKRKREAD	11.0		12–21	Yes	All VP proteins	108–117
	AalDV1	RGTKRKRETG	10.0		14–23	Yes		111–120
	AaeDV1	RGTKRKRDAG	10.0		14–23	No		
	AaeDV2	RGTKRKRGAG	10.0		14–23	Yes		111–120
	AgDV, CppDV	RGTKRKRGAE	11.0		14–23	Yes		109–118
*Penstyldensovirus*		Described in Owens et al., 2013 [27].				No		
*Hepandensovirus*		Described in Owens et al., 2013 [27].				Yes	VP ORF	174–181

* Provided are high-score NLS motifs, if not indicated otherwise. ** Indicated are proteins for which the expression system has been interpreted, or VP-coding ORFs which contain the corresponding NLS/NES motif. *** Amino acid positions are provided in accordance with the translated amino acid sequences for the corresponding VPs in cases in which the expression strategy is known or for the VP coding ORF in other cases, as they are provided in the corresponding virus NCBI GenBank entries. ^#^ Score value is provided for the bipartite motif (in parenthesis). ^##^ No score values were obtained since motifs were predicted by Wolf PSORT.

## Supplementary Materials

The following are available online at http://www.mdpi.com/1999-4915/10/7/370/s1, Figure S1: Indirect immunofluorescence of Cos-1 cells transiently expressing the capsid BgDV1 VP3 protein fused with GFP.
