# Supplementary material for: Intracellular Localization of Blattella germanica Densovirus (BgDV1) Capsid Proteins
Source: Viruses. 2018 Jul 14;10(7):370. doi: 10.3390/v10070370 (PMC6071259; doi:10.3390/v10070370)
Supplement: Supplementary file 1 [file viruses-10-00370-s001.zip › viruses-314537-supplementary.pdf]

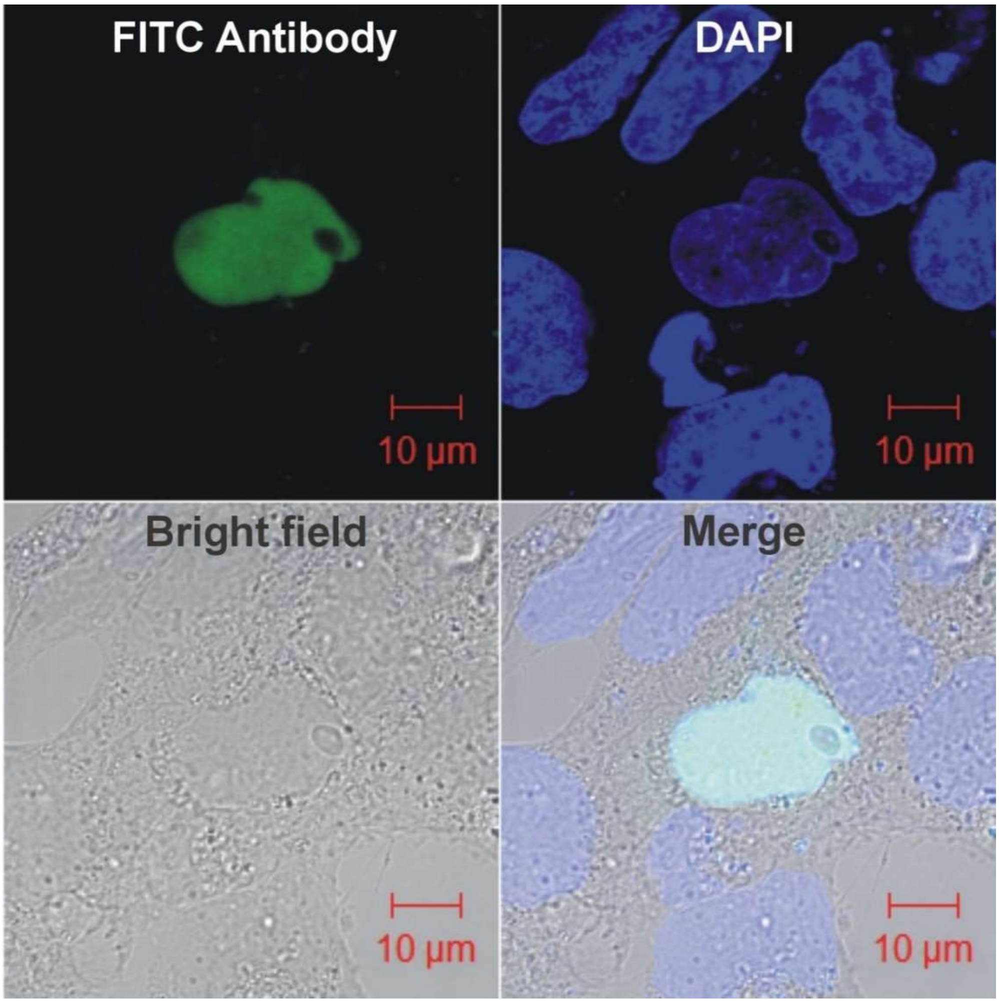

Figure S1. Indirect immunofluorescence of Cos-1 cells transiently expressing the capsid BgDV1 VP3 protein fused with GFP. The transfected Cos-1 cells transiently expressing N-fused with GFP VP3 protein were fixed in 4% PFA and subsequently immunostained with both primary rabbit antibodies against the C-end part of VP1 capsid protein and goat anti-rabbit FITC-conjugated secondary antibody. Cell nuclei were counterstained with DAPI. The intracellular localization of the fluorescent signals was analyzed by confocal microscopy.
